# Effect of Interpregnancy Interval on Pregnancy Outcome in a Tertiary Care Centre: A Descriptive Cross-sectional Study

**DOI:** 10.31729/jnma.8775

**Published:** 2024-10-31

**Authors:** Poonam Koirala, Ishita Koirala, Sunita Pun, Shweta Karna

**Affiliations:** 1 Department of Obstetrics and Gynaecology, Tribhuvan University Teaching Hospital, Maharajgunj, Kathmandu, Nepal; 2 Nepal Medical College and Teaching Hospital, Jorpati, Kathmandu, Nepal

**Keywords:** *interpregnancy interval*, *preterm birth*, *short interpregnancy interval*

## Abstract

**Introduction:**

Interpregnancy interval has a major impact on the maternal and fetal health worldwide. Women with short interpregnancy interval show higher rates of low birth weight and preterm babies. So this study aimed to study the effect of interpregnancy interval on pregnancy outcomes.

**Methods:**

This descriptive cross-sectional study was conducted in the Department of Obstetrics and Gynaecology of a tertiary care center after taking ethical approval from Institutional Review Committee (Reference number: 229/080/081(6-11)E2). Data from September 1^st^, 2023 to January 30^th^, 2024 was collected. All multigravida women with singleton pregnancy after 28 weeks of gestation who delivered in our hospital with previous birth were enrolled in the study. Point estimate and 95% Confidence Interval were calculated. The data was entered and analyzed using IBM SPSS Statistics version 26.0

**Results:**

Among the women, 31 (3.57%) women had short interpregnancy interval ≤18 months whereas 836 (96.43%) had interpregnancy interval of >18 months. The mean age of the women was 28 years. Previous cesarean section was common indication of cesarean section 27 (87.09%) among women with short interpregnancy interval. These women had higher rates of preterm and low birth weight babies 11(35.58%) and 15(48.38%).

**Conclusions:**

Women with short interpregnancy interval ≤18 months had higher preterm births and low birth weight babies. Pregnancy induced hypertension, hypothyroidism and gestational diabetes were increased in women with interpregnancy interval of >18 months.

## INTRODUCTION

Pregnancy and childbirth have always been the major events in a woman life. World Health Organization (WHO) has recommended interval of 24 months before attempting the next pregnancy and 33 months before the next birth to promote healthier maternal and child health outcomes.^1^ A short interpregnancy interval (SIPI) is a period between delivery of the previous infant and conception of the current pregnancy of less than or equal to 18 months.^2^

Studies have examined the association between interpregnancy interval(IPI) and adverse pregnancy outcomes like small for gestational age birth,^3^ preterm birth,^4^ low birth weight,^5^ child mortality^6,7^ and maternal death.^8^ Literature also supports the association of SIPI with risk of PROM,^9,10^ placenta praevia^11^ and uterine rupture for women who delivered by cesarean section^12,13^ whereas long IPI >18 months with hypertension. All these morbidities necessitate optimal IPI for childbirth and prompts that studies like this are necessary. This study aimed to study IPI's effect on the pregnancy outcome.

## METHODS

This descriptive retrospective cross-sectional study was conducted in the Department of Obstetrics and Gynaecology of Tribhuvan University Teaching Hospital, Maharajgunj, Kathmandu, Nepal after obtaining ethical clearance from the Institutional Review Committee (Reference number:229/080/081 (6-11)E2). Data from September 1^st^ 2023 to January 30^th^ 2024 was collected between 1^st^ February to 30^th^ February 2024. Total population sampling technique was used. All multigravida women with singleton pregnancy after 28 weeks of gestation who delivered in our hospital with previous birth irrespective of outcome were enrolled in the study. Primigravida, multigravida with history of previous miscarriages without any previous birth and women with multifetal pregnancy were excluded from the study. The IPI was calculated as the time elapsed between the woman's last delivery and the date of the last menstrual period and cases were categorized as ≤18 months and > 18 months.^2^ Low birth weight babies were taken as birth weight of less than 2.5 kg as defined by WHO (2004).^1^ Preterm delivery was taken as delivery less than 37 weeks of gestation from the last menstrual date. The study variables included age, parity, gestational age, placenta previa (PP), abruptio placentae, scar rupture, pre-eclampsia, gestational diabetes (GDM), mode of delivery, postpartum haemorrhage (PPH), low birth weight babies, preterm births, intrauterine growth retarded babies and stillbirth. The detailed information was collected from the patients file, partograph, and neonatal record book. The data was entered and analyzed using IBM SPSS Statistics version 26.0.

## RESULTS

There were total 1561 deliveries during study period of 6 months. Out of them 694 (44.45%) women including both primigravida and multigravida with history of prior miscarriages and without any previous birth were excluded. Thus, a total of 867 (55.55%) cases were included in the study. Among the total deliveries that included in the study, 31 (3.57%) women had short IPI ≤18 months whereas 836 (96.43%) had interpregnancy interval of >18 months. The mean age of the women was 28±4.2 years.

Amongst the patients with an IPI interval ≤ 18 months 17 (54.83%) were in the age group of 20-25 years, 12 (38.70%) were in the age group of 26-30 years and 2 (6.45%) were in the age group of 31-35 years. As per parity, amongst the patients with an IPI interval ≤18 months, 24 (77.5%) patients were with parity 1, 5 (16.13%) patient were with parity 2 and 1 (6.45%) patient was with parity ≥3. Amongst the patients with an IPI interval > 18 months 225 (26.91%) patients belonged to the age group of 20-25 years and 33 (3.94%) patients were >35 years. Among the patients with IPI > 18 months 202 (24.16%) were with parity 1, 115 (13.75%) patients had parity ≥3 (Table 1).

**Table 1:** Maternal characteristics in relation to interpregnancy interval (n= 867).

Age	IPI ≤ 18 months (n=31) n (%)	IPI > 18 months (n=836) n (%)
≤19	-	-
20-25	17 (54.83)	225 (26.91)
26-30	12 (38.70)	427 (51.07)
31-35	2 (6.45)	151 (18.06)
>35	-	33 (3.94)
**Parity**		
1	24 (77.5)	202 (24.16)
2	5 (16.13)	519 (62.08)
≥3	1 (6.45)	115 (13.75)

IPI = Inter pregnancy interval

Among the patients with IPI ≤ 18 months, vaginal delivery was done in 4 (12.90%) patients and cesarean section was done in 27 (87.09%) patients. Out of total patients with IPI ≤ 18 months 2 (6.45%) had PPH. Similarly, 1 (3.22%) PP, 1 (3.22%) GDM, 3.22 PIH and 4 (1.10%) preterm births was seen amongst patients with IPI ≤ 18 months. Proportion of mode of delivery and outcome in groups IPI ≤ 18 months and IPI > 18 months is given below (Table 2).

**Table 2:** Maternal characteristics in relation to IPI (n = 867).

Characteristics	IPI ≤ 18 months (n=31) n (%)	IPI >18-59 months (n=836) n (%)
**Mode of delivery**		
Vaginal	4 (12.90)	359 (42.94)
Cesarean section	27(87.09)	461 (55.14)
Instrumental delivery		
Vaccum	-	16 (1.91)
Forceps delivery	-	-
**Outcome**		
Preterm birth	11 (35.48)	133 (15.9)
PPH	2 (6.45)	8 (0.95)
Pregnancy induced hypertension	1 (3.22)	58 (6.93)
GDM	1 (3.22)	38 (4.54)
Obstetric cholestasis	-	8 (0.95)
Heart disease	-	20(2.39)
Hypothyroidism	-	45 (5.38)
Antiphospholipid syndrome	-	4 (0.47)
APH	-	13 (1.55)
PP	1 (3.22)	6 (0.71)

IPI = Inter pregnancy interval, GDM = Gestational diabetes mellitus, APH = Antepartum hemorrhage, PP = Placenta previa, PPH = Post partum hemorrhage

Regarding indications of cesarean section, among the patients with SIPI, previous cesarean section was an indication in 4 (14.81%) patients. Previous cesarean section with scar tenderness was indication of cesarean section among 8 (29.62%) patients with SIPI. Among the patients with IPI > 18 months previous cesarean section was an indication of cesarean section in 187 (22.36%) patients. Fetal distress was an indication for cesarean in 103 (12.32%) patients with IPI > 18 months group and it was an indication of cesarean section in 2 (7.47%) patients with IPI ≤ 18 months group (7.47%) (Table 3).

**Table 3:** Indications of cesarean sections with SIPI ≤ 18 months and IPI more than 18 months (n = 867).

Indications of cesarean sections	IPI ≤ 18 months (n=27) n (%)	IPI > 18 months (n=461) n (%)
Previous cesarean section	4 (14.81)	187 (22.36)
Previous cesarean section with scar tenderness	8 (29.62)	14 (1.67)
Previous cesarean section with thin scar <2.5 mm	6 (22.22)	5 (0.59)
Previous cesarean section with Intrauterine growth retardation	4 (14.81)	15 (1.79)
Previous cesarean section with placenta previa with BOH	1 (3.7)	6 (0.71)
Fetal distress	2 (7.47)	103 (12.32)
APH	-	12 (1.43)
IUGR	3 (11.11)	13 (1.55)
Malpresentation	-	13 (1.55)
Cord prolapse	-	2 (0.23)
Severe oligohydromnios	-	20 (2.39)
Non progress of labour	-	14 (1.67)
subfertility	-	4 (0.47)
Decreased fetal movement	-	16 (1.91)
Failed induction of labour	-	37 (4.42)

A total of 11 (35.48%) of women with SIPI had preterm births whereas 133 (15.90%) of women with IPI of >18 months had preterm births. Fifteen (48.38%) of women with a short interpregnancy interval had low birth weight babies whereas 115 (13.75%) of women with IPI of >18 months had low birth weight babies (Table 4).

**Table 4:** Fetal characteristics in relation to IPI (n= 867).

Fetal parameters	IPI ≤ 18 months (n=31) n (%)	IPI > 18 months (n=836) n (%)
Low birth weight babies	15 (48.38)	115 (13.75)
Preterm birth	11 (35.48)	133 (15.90)
Intrauterine growth retardation	6 (19.35)	16 (1.91)
Still birth	-	1 (0.11)

## DISCUSSION

In our study, low birth weight babies 15 (48.38%) was the most common fetal characteristic in relation to IPI seen among women with IPI of ≤18 months followed by preterm birth 11 (35.48%) and intrauterine growth retardation 6 (19.35%). Similarly, preterm birth 133 (15.90%) was the most common fetal characteristic in women with IPI > 18 months followed by low birth weight babies 115 (13.75%), intrauterine growth retardation 6 (19.35%) and stillbirth 1 (0.11%). Similar findings are showed in a study done in Lucknow, India where preterm births, low birth weight and intrauterine growth retarded babies were found in higher proportion in IPI ≤ 24 months than group >24 months (24.13%, 33.33%, 9.19% vs 10.4%, 14.4%, 4%) respectively.^25^ However the proportion of preterm and low birth weight 11 (35.48%) 15 (48.38%) among women with SIPI are slightly higher in present study in contrast to the above study. This variation may be due to variation in the population studied, ethnicity and the associated various medical conditions and also due to limited data of women with SIPI. However in the present study both short and IPI > 18 months showed a high rate of preterm births in comparison to various studies. This also may be explained by the associated medical disorders like diabetes, hypertension, preeclampsia, thyroid disorders and others medical conditions with advancing age. This study was conducted in a multidisciplinary referral tertiary care center where many referred cases with lots of medical conditions arrive for delivery.

Majority of patients with SIPI underwent cesarean section in the present study due previous cesarean section, followed by those with scar tenderness and thin scar. This finding is quite similar to a study done in New Delhi, India^20^ where SIPI had higher proportion of women with history of previous cesarean section 85.7% and they showed scar tenderness which was statistically significant. Inadequate time period for the scar to heal in SIPI may be the reason for the thin scar or scar tenderness in patients with SIPI with previous cesarean section.

Timing between pregnancies is very crucial for the nutritional wellbeing of the mother, but generally little emphasis is given on birth spacing in developing countries. Certain factors are always considered whenever couple decide to get pregnant like their age, cultural aspects, religious beliefs, financial status and previous pregnancy outcome however nutritional well being of the woman is never considered especially in developing countries.

Shorter interpregnancy intervals increase risks of maternal, infant and child mortality due to “maternal depletion syndrome”, where inadequate time between pregnancies hinders replenishment of vital nutrients.^14^ Conversely long IPI have been found to be associated with increased risk of pre-eclampsia^15^ and labour dystocia.^16^ Thus IPI has deleterious effect on the pregnancy outcome, especially the short IPI, where the health status of the mother is deprived due to lack of proper nutrition and stress of the previous pregnancy. Breastfeeding requires extra energy and stores which usually are not replenished adequately during a short interpregnancy interval(SIPI). A study by Klerman and coworkers reported that women with SIPI and poor nutritional status had adverse pregnancy outcomes.^4^

Short IPI was present in 31(3.57%) patients of total deliveries that took place in the present study which is quite low in comparison to study conducted in Ethiopia^17^ where the prevalence was 40.9%. Similar study done in India^18^ showed prevalence of SIPI of 25%. However the time period taken in our study was 18 months from previous birth to the conception of index pregnancy, whereas in these studies it was taken as 24 months. Various other factors also affect the SIPI such as non exclusive breastfeeding, poor nutritional status, desire of male child, previous pregnancy outcome and method of contraceptive used, study period and sample size.

In the present study, a total of 11 (35.48%) of women with SIPI had preterm births and 15 (48.38%) women had low birth weight babies. Similar results were shown by a study done in Tanzania,^19^ where mothers having SIPI had more risk of low-birth weight babies and perinatal death. A study conducted in New Delhi, India showed even higher proportion of preterm 62.50% and low birth weight babies 76.47% in women with SIPI ≤ 18 months^20^ in comparison to present study. Short IPI is associated with maternal iron and folic acid depletion which is also linked with increased risks of preterm birth, low birth weight and growth restrictions.^21^ Similar findings are seen in studies done in B.P Institute of health sciences Dharan, Nepal,^22^ Northern Tanzania^19^ and Haryana, India.^23^ Women do not get adequate time to shed extra weight which she gains during pregnancy. Maternal overweight or obesity may result in increased risk of inflammatory up-regulation and increased levels of inflammatory proteins which lead to cervical ripening and cause weakening of membranes and preterm myometrial contractions through prostaglandin activation.^24^

In present study pregnancy induced hypertension was seen in 58 (6.93%) of women with IPI more than 18 months followed by hypothyroidism in 45 (5.38%) and gestational diabetes mellitus in 38 (4.54%). This is in-contrast to study from USA^25^ where GDM is seen in increased proportion in SIPI, but actually as the age advances the risk of GDM becomes higher with the increasing body mass index. A study conducted in India, Lucknow^26^ stated that severe pre-eclampsia and eclampsia were more in interpregnancy interval ≤ 24 months than group IPI > 24 months (8%, 4.6% vs 4.0%, 2.4%) respectively. However the present study shows an increased proportion of pregnancy induced hypertension among women with IPI > 18 months. This finding is quite similar with study conducted in New Delhi, India where interpregnancy interval between 1959 months showed higher rates of pregnancy induced hypertension 78.6%.^20^ Similarly study conducted in Mumbai, India^27^ also showed association of longer IPI with hypertension. Heart disease, obstetric cholestasis and antiphospholipid syndrome were also found to have increased among women with IPI >18 months in present study. All this is attributed to the change in lifestyle and dietary habits with advancing age among the population studied and the ethnicity.

We should aim at reducing the primary cesarean section as the majority of women with short IPI presented with indications as previous cesarean section followed by scar tenderness and thin scar in the present study. Proper counseling should be given at the time of discharge regarding family planning. These are the women who need special attention as the risk of scar rupture or scar dehiscence always exists. Adverse fetomaternal outcome is associated with short inter pregnancy interval, thus counseling regarding birth spacing and family planning is must at the time of discharge after delivery. Clinical postpartum practice^28,29^ and public health^1^ guidelines also recommend interpregnancy intervals of 18-24 months.

Modification of interpregnancy interval can always be done with the help of implementing various national health policies regarding family planning and its resources. National health training of service providers on family planning may further help in approaching the goal of optimal delivery interval between the pregnancies. Thus we should focus on reduction of primary cesarean section and implementation of various strategies regarding postpartum contraception counselling and interpregnancy care.

There are certain limitations of this study, being single centered and small sample sized. Due to this, the findings can not be generalized. Large sample sized, randomized studies should be conducted on the IPI to evaluate the best interval for the fetal and maternal well being. Prospective study with categorization done into three categories: SIPI, suboptimal IPI and IPI longer than 59 months could have given us better results with large sample size. Another limitation of this study is the significant discrepancy in sample sizes between the comparison groups, which may have influenced the results. The smaller sample size in one group, particularly for the women with IPI ≤ 18 months may have limited the statistical power and robustness of the findings.

## CONCLUSIONS

In our study, low birth weight babies was the most common fetal characteristic in relation to IPI seen among women with IPI of ≤ 18 months followed by preterm birth and intrauterine growth retardation. Similarly, preterm birth was the most common fetal characteristic in women with IPI >18 months followed by low birth weight babies and intrauterine growth retardation. Previous cesarean section with scar tenderness was the commonest indication of cesarean section with SIPI, whereas pregnancy induced hypertension followed by hypothyroidism and gestational diabetes mellitus were higher among women with IPI longer than 18 months. However the differences seen between two groups may be due to limited data of SIPI.
